# Curcumin-Loaded Mesoporous Silica Nanoparticles Markedly Enhanced Cytotoxicity in Hepatocellular Carcinoma Cells

**DOI:** 10.3390/ijms20122918

**Published:** 2019-06-14

**Authors:** Zwe-Ling Kong, Hsiang-Ping Kuo, Athira Johnson, Li-Cyuan Wu, Ke Liang B. Chang

**Affiliations:** Department of Food Science, National Taiwan Ocean University, Keelung 20224, Taiwan; williamkuococo@hotmail.com (H.-P.K.); athirajohnson07@gmail.com (A.J.); ninec9@hotmail.com (L.-C.W.); klchang@mail.ntou.edu.tw (K.L.B.C.)

**Keywords:** curcumin, silica, chitosan, nanoparticles, anti-tumor, antioxidant activity

## Abstract

Curcumin, a natural polyphenol extracted from a perennial herb *Curcuma longa* has been verified for many physiological activities such as anti-oxidant, anti-inflammatory, and anti-tumor properties. The direct use of curcumin cytotoxicity studies are limited due to its unstable chemical structure, low bioavailability, easy oxidation, and degradation by ultraviolet (UV) light etc. Trying to overcome this problem, silica-encapsulated curcumin nanoparticles (SCNP) and chitosan with silica co-encapsulated curcumin nanoparticles (CSCNP) were prepared by silicification and biosilicification methods, respectively, and encapsulated curcumin within it. We investigated the antitumor properties of SCNP and CSCNP on different tumor cell lines. Scanning electron microscopy (SEM) analysis revealed that both SCNP and CSCNP were almost spherical in shape and the average particle size of CSCNP was 75.0 ± 14.62 nm, and SCNP was 61.7 ± 23.04 nm. The results show that CSCNP has more anti-oxidant activity as compared to curcumin and SCNP. The higher cytotoxicity towards different cancerous cell lines was also observed in CSCNP treated tumor cells. It was noted that the SCNP and CSCNP has a high percentage of IC_50_ values in Hep G2 cells. The encapsulation of curcumin improved instability, antioxidant activity, and antitumor activity. Our results demonstrated that nanoencapsulation of curcumin with silica and chitosan not only increase curcumin stability but also enhance its cytotoxic activity on hepatocellular carcinoma cells. On the basis of these primary studies, the curcumin-loaded nanoparticles appear to be promising as an innovative therapeutic material for the treatment of tumors.

## 1. Introduction

Cancer is the second leading cause of mortality in the world and approximately 1,665,540 people in the United States suffered from cancer by 2014 [1]. A tumor (neoplasm) is an uncontrolled growth of cells and becomes less responsive to normal growth control. Invasion and metastasis are the major features of a tumor and is categorized into benign and malignant tumors. Benign tumors are non-cancerous and they will not spread to other areas. Malignant tumors are cancerous and can spread to other tissues (metastasis) via the bloodstream and lymph nodes [2]. Tumors may occur in any part of the body including the skin, lungs, bone, intestines, and breast etc. Uncontrolled proliferation, induction of angiogenesis, active invasion, metastasis, immortality, and evasion of growth suppressors are the major traits of cancerous cells [3]. Apoptosis is an ordered cell death mechanism involving many complex pathways. It is a key mechanism to eliminate damaged cells and control cell proliferation. The processes of apoptosis involve the shrinkage of cells, chromatin condensation, membrane blebbing, and deoxyribonucleic acid (DNA) fragmentation [4,5]. The resistance of tumor cells occurs due to the defective apoptosis signaling pathway by mutation [4]. During initiation, the oncogene is activated and the processes of oncogenesis leads to the formation of cancerous cells. In most cases, tumors are associated with *p53* gene mutation and it became known as the first tumor suppressor gene linked to apoptosis [6]. The primary trial of each chemotherapeutic drug is based on its potential cytotoxicity towards the cancer cell lines. A decrease in cell numbers over time is an important requirement for an in vitro cytotoxicity assessment [7]. Currently, the antitumor drug designs are based on their selective targeting towards tumor cells. This will be achieved by caspase activation, phosphatidylserine exposure, and poly (ADP-ribose) polymerase (PARP) cleavage [8]. The conventional treatments such as radiation and chemotherapy have not been widely recommended because of their side effects. 

The emergence of nanotechnology has changed the conventional concepts and ideas of the pharmaceutical fields. Mesoporous silica nanoparticles (MSNs) were first introduced by Mobil corporation scientists in 1992. They have a unique mesoporous structure with high chemical stability, low toxicity, high drug loading capacity, controlled release, biocompatibility, high surface area, target delivery, large pore volume, and surface functionality [9]. The passive target of nanoparticles in cancer therapies is achieved because of the enhanced permeability and retention (EPR) effect of the cancerous cells. The impaired lymphatic system and defective vascular architecture allow the nanoparticles to enter into the cancerous cells. The MSNs are internalized into the cells via phagocytosis and pinocytosis [10]. Polypeptides and polysaccharides are responsible for the formation of biosilica via the repeated phase-separation mediated templating mechanism and the aggregation-based mechanism. Chitosan is a cationic polysaccharide having a terminal amino group has been proven to facilitate silicification through catalyzing the hydrolysis/condensation of the silica source and the subsequent aggregation of silica [11]. A recent study shown a targeted delivery of calcium leucovorin galactosylated chitosan-functionalized mesoporous silica nanoparticle to treat colon cancer. The surface of the MSNs contains a large number silanol groups, which allow easy functionalization, controlled drug release, and drug loading [12]. Chitosan is obtained from the deacetylation of chitin. It is composed of β-(1,4)-linked glucosamine units (2-amino-2-deoxy-β-d-glucopyranose) and N-acetylglucosamine units (2-acetamino-2-deoxy-β-d-glucopyranose) in different ratios [13]. The amino group on chitosan provide controlled release, permeation enhancement, mucoadhesion, in situ gelation etc. [14]. P^H^ responsive delivery of curcumin from chitosan mesoporous silica nanoparticles were reported by Nasab et al., 2018 [15]. Cytotoxicity assays revealed IC_50_ after 72 h treatment with free curcumin and curcumin-loaded nanoparticles on U87MG glioblastoma cancer cell line were 15.20 and 5.21 μg/mL (*p*  < 0.05). respectively [15]. 

Curcumin (1, 7-bis (4-hydroxy-3-methoxyphenyl)-1, 6-heptadiene-3, 5-dione) is a yellow colored polyphenol obtained from turmeric (*Curcuma longa*) that has been known for thousands of years for its pharmacological activities [16]. It has a wide spectrum of therapeutic activities including anti-inflammatory, antioxidant, antitumor, antiviral, antimicrobial, and analgesic effects etc. It is insoluble in water but soluble in ethanol and acetone. The anti-inflammatory property of curcumin is carried out by blocking the IκK-mediated phosphorylation and degradation of IκBα. As a consequence, the nuclear factor (NF)-κB will bind to the IκBα and does not induce transcription [17]. The growth, invasion, and metastasis of the cancerous cells can be prevented by curcumin via interfering with their proliferation process [18]. Curcumin scavenges superoxide, nitric oxide, and hydrogen peroxide radicals and reduces the inflammation by lowering the histamine levels and produce an inflammatory response to cytokines [19]. Curcumin also induces apoptosis through the inhibition of cyclooxygenase (COX)–2 and affects various growth factor receptors and molecules involved in tumor growth, angiogenesis, and metastasis [20]. It was understood that curcumin causes cell cycle arrest at the G0/G1 phase and S and G2/M phases in leukemic cells and breast cancer cells respectively [21]. Apart from this, curcumin moderates transcription factors downregulates cytokines and inhibits the activity of c- Jun N-terminal kinase, protein tyrosine kinases and protein serine/threonine kinases of a wide variety of tumor cells and cancer stem cells [22]. The mode of action of curcumin is different in each type of cells. A recent study showed that miR-21/PTEN/Akt signaling pathway is the key mechanism of the anti-cancer effects of curcumin on breast cancer cells [23]. Reduction in vascular endothelial growth factor (VEGF) expression and PI3K/AKT signaling were noticed in hepatocellular carcinoma model [24]. Curcumin inhibited zeste homolog 2 (EZH2) in lung cancer cells both transcriptionally and post-transcriptionally, thereby decreasing the expression of NOTCH1 [25]. The process of nanoencapsulation enhances the site-specific activity and optimizes the therapeutic efficacy of curcumin [26]. Recent study showed that the curcumin-loaded nanoliposomes (Cur-NLs) protected the tetrachloromethane- (CCl_4_^-^) induced liver injury in mice. Cur-NLs attenuated the hepatic necrosis and decreased the malonaldehyde (MDA) level [27]. Another study demonstrated the inhibition of the growth and *hTERT* gene expression in human breast cancer cells by nano-encapsulated metformin-curcumin in poly (lactic-co-glycolic acid)/polyethylene glycol (PLGA/PEG) [28]. 5-flurouracil and curcumin loaded N,O-carboxymethyl chitosan nanoparticles 4 showed a sustained release and enhanced anti-cancer effects both in vitro and in vivo [29]. Song et al., 2018 [30] reported the high uptake efficiency, toxicity, and sustained release in human Caucasian breast adenocarcinoma cells (MDA-MB-231). It supported the dose-depended delivery of curcumin on cancer cells [30]. Higher toxicity of PEGylated curcumin nanoparticles (IC_50_ = 4.2 μM) than the free curcumin at all doses were observed in CT-26 cells with an 8-fold decrease in the half-maximal inhibitory concentration (IC_50_) values of the free Cur (IC_50_ = 33.4 μM) after 24 h [31]. There is a significant difference between curcumin and nanocucumin effects on growth depression of on human breast adenocarcinoma cell line (MDA-MB231) (*p* < 0.01). The IC_50_ curcumin after 24 h, 48 h and 72 was 79.58 μg/mL and 53.18 μg/mL and 30.78 μg/mL whereas this value for nanocurcumin was 37.75 μg/mL and 23.25 μg/h and 12.99 μg/mL, respectively (*p* < 0.01) [32]. A decreased proliferation of esophageal squamous cell carcinoma (KYSE-30) cells was observed after treatment with nanocurcumin (71.09%) without affecting the normal cells. In addition to this, it down-regulated the expression of cyclin D1 [33]. In vitro models of the toxicity studies have diverse application in the selection of cancerous cells and tumor microenvironments. The cancer cells grow easily and facilitate the direct comparison between the results under in vitro conditions [34]. The direct use of curcumin is limited due to its low water solubility, poor chemical stability, and low oral bioavailability [35]. Along with this, research based on the cytotoxicity of curcumin in various cancer cells are still rare. The present study examined the cytotoxic effect of curcumin- loaded mesoporous silica nanoparticles (MSNs) on different tumor cell lines together with an examination of curcumin parameters after storage. 

## 2. Results

### 2.1. Characterization of Silica-Encapsulated Curcumin Nanoparticles (SCNP) and Chitosan with Silica Co-Encapsulated Curcumin Nanoparticles (CSCNP)

**Figure 1:**
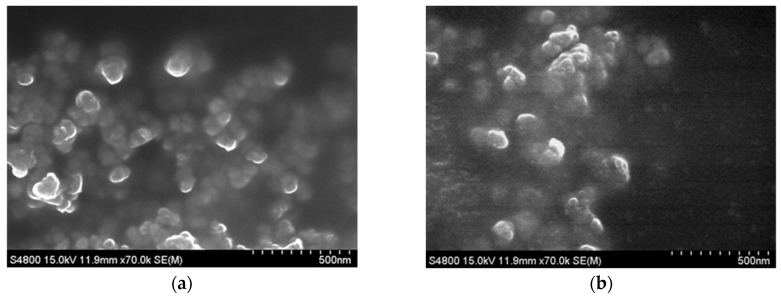
Scanning electron microscope (SEM) images of (**a**) CSCNP and (**b**) SCNP. SCNP: silica-encapsulated curcumin nanoparticles; CSCNP: chitosan with silica co-encapsulated curcumin nanoparticles.

Silica-encapsulated curcumin nanoparticles (SCNP) and chitosan with silica co-encapsulated curcumin nanoparticles (CSCNP) were prepared by silicification and biosilicification methods respectively. The nanoparticles were characterized by scanning electron microscopy (SEM) and the dynamic light scattering (DLS) method. From SEM images, CSCNP were relatively spherical in shape with uniform size distributions as compared to SCNP (Figure 1). The average size of CSCNP was 75.0 ± 14.62 nm and SCNP was 61.7 ± 23. 04 nm. The sizes of nanoparticles were also confirmed by DLS analysis. The average particle sizes of SCNP and CSCNP were 111.0 ± 2.95 nm and 112.8 ± 3.00 nm, respectively (Table 1). The difference in the particle sizes between SEM and DLS was due to the dryness of the particle during SEM sample preparation. Nanoparticle-based drug carrier system increase the bioavailability of the drug in the targeted site. Literature showed that a particle between the size of 100 to 1000 nm can enter into the cancer cells instead of normal cells because of the EPR effect [36]. 

### 2.2. Antioxidant Activities of SCNP and CSCNP

**Figure 2:**
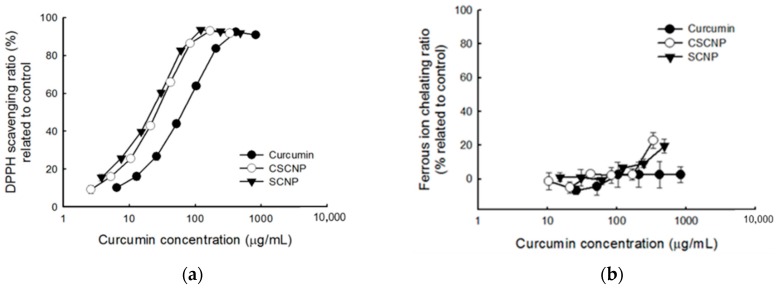
Antioxidant activities of curcumin, SCNP, and CSCNP. (**a**) DPPH scavenging activity and (**b**) ferrous ion chelating activity of curcumin, SCNP and CSCNP. Data were shown by mean ± standard deviation (SD) of 3 independent experiments (*n* = 3) with 3 technical replicates. DPPH: 2, 2-diphenyl-1-picrylhydrazyl; SCNP: silica-encapsulated curcumin nanoparticles; CSCNP: chitosan with silica co-encapsulated curcumin nanoparticles.

The imbalance between the production of reactive oxygen species (ROS) and antioxidants generates oxidative stress within the cells. Under stress conditions, the cell structure is damaged and cell functions are altered. [37]. The antioxidant activities of SCNP and CSCNP were evaluated by 2, 2-diphenyl-1-picrylhydrazyl (DPPH) radical scavenging activity (Figure 2a) and ferrous ion chelating activity (Figure 2b). The DPPH radical scavenging activity increased with increasing concentrations of curcumin and nanoparticles. As compared to pure curcumin and SCNP, the CSCNP showed slightly better activity towards the DPPH radical scavenging and the half maximal effective concentration (EC_50_) of curcumin, CSCNP and SCNP were 59 μg/mL, 32 μg/mL and 44 μg/mL, respectively (Figure 2a). Interestingly, curcumin has no ferrous ion chelating activity but both SCNP and CSCNP exhibited better activity at higher concentration (Figure 2b). Noting that curcumin itself does not have the property to chelate ferrous ion and CSCNP has better activity than SCNP indicated the involvement of chitosan in chelating ferrous ions. A previous study reported that the stimulation of ROS production is also a pro-apoptotic action of curcumin to induce cell death [38]. These results suggest that curcumin nanoparticles might also influence the ROS levels through free radicals scavenging or ferrous iron chelation. The increased antioxidant of nanoencapsulated curcumin (scavenging capacity, SC_50_ = 13.9 μg/mL) than free curcumin (SC_50_ = 16.7 μg/mL) by DPPH radical scavenging activity were reported by Huang et al., 2016 with the differences statistically significant at *p* < 0.05 [39]. 

### 2.3. Cytotoxicity of SCNP and CSCNP

**Figure 3:**
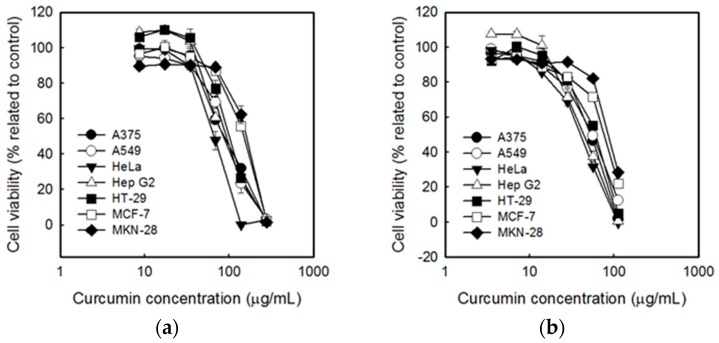

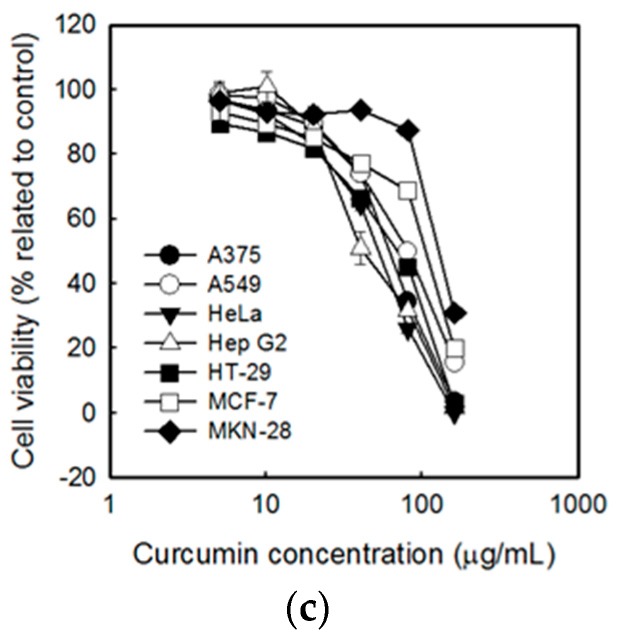
Effects of (**a**) curcumin, (**b**) CSCNP, and (**c**) SCNP on cancer cell viability. The cell number for 7 kinds of cells were adjusted to 2 × 10^5^ cells/mL and treated with different concentrations of samples for 48 h. The cell viability was analyzed by MTT assay. Data were shown by mean ± SD of 3 independent experiments (*n* = 3) with 3 technical replicates. SCNP: silica-encapsulated curcumin nanoparticles; CSCNP: chitosan with silica co-encapsulated curcumin nanoparticles.

Tumor cells are characterized by the uncontrolled proliferation of cells [40]. Regulations of the differentiation of cells are needed to control tumor growth. The non-toxicity of curcumin on normal cells where described elsewhere [41,42]. The cytotoxicy of curcumin (Figure 3a), SCNP (Figure 3c) and CSCNP (Figure 3b) were examined by performing the 3-(4,5-dimethylthiazol-2-yl)-2,5-diphenyltetrazolium bromide (MTT) assay against seven types of tumor cell lines. Figure 3a indicated that curcumin has significant toxicity towards the cancerous cells and the cell viabilities of each cell were decreased with the increase in concentrations. The similar results were also observed in SCNP and CSCNP treated cells. It was noticed that human cervical squamous carcinoma cell line HeLa was more sensitive to curcumin, SCNP and CSCNP. The human breast carcinoma cell line MCF-7 and the human gastric adenocarcinoma cell line MKN-28 were more tolerant towards curcumin and nanoparticles. The comparison of the IC_50_ value of each sample was listed in Table 2. IC_50_ values of CSCNP were lower than that of the curcumin and SCNP. It was also noted that the most significant difference between the percentage of IC_50_ values of curcumin and nanoparticles was observed in Hep G2 cells. About 49% (CSCNP) and 54% (SCNP) in IC_50_ value in Hep G2 cells indicated that both CSCNP and SCNP were more toxic to HepG2 cells than free curcumin. Consequently, further analyses were carried out using Hep G2 cells. A previous study reported the increased bioavailability of curcumin after encapsulation and both free and nanoencapsulated curcumin suppress COX-2 and VEGF expression and thereby reduced the proliferation of hepatocellular carcinoma cells [43]. It was also noted that curcumin cause toxicity via disturbing the cell homeostasis and affect cell function including intracellular free Ca^2+^ concentration and mitochondrial membrane potential in HepG2 cells [44].

### 2.4. Cytotoxicity of SCNP and CSCNP against Hep G2 Cells

**Figure 4:**
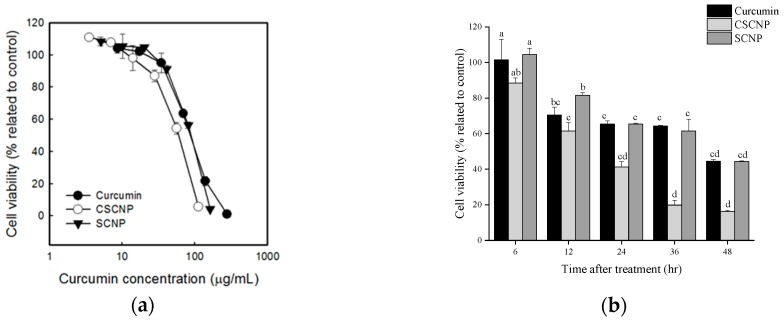
MTT assay of Hep G2 cells after different curcumin or nanoparticle dosages and time durations. The cells were treated with samples for (**a**) 48 h at different dosages and (**b**) 100 μg/mL at different time intervals. The initial cell number was adjusted to 2 × 10^5^ cells/mL. Data were shown by mean ± SD of 3 independent experiments (*n* = 3) with 3 technical replicates. SCNP: silica-encapsulated curcumin nanoparticles; CSCNP: chitosan with silica co-encapsulated curcumin nanoparticles.

Cytotoxicity of curcumin, SCNP, and CSCNP against Hep G2 cells were evaluated by 1-(4,5-Dimethylthiazol-2-yl)-3,5-diphenylformazan (MTT) assay. From Figure 4a, it was understood that the cell viability of Hep G2 cells dropped gradually after the cells were treated with curcumin and nanoparticles. As compared to curcumin and SCNP, the CSCNP showed more reduction in cell viability of cancerous cells at lower concentrations. The cell viability was also analyzed in a time-dependent manner (Figure 4b). The cell viability of Hep G2 cells was significantly reduced with CSCNP treatment when compared to other groups at different time intervals. From Figure 4, it was understood that CSCNP was more efficient in cytotoxicity of Hep G2 than curcumin and SCNP.

### 2.5. Lactate Dehydrogenase (LDH) Leakage Assay

**Figure 5:**
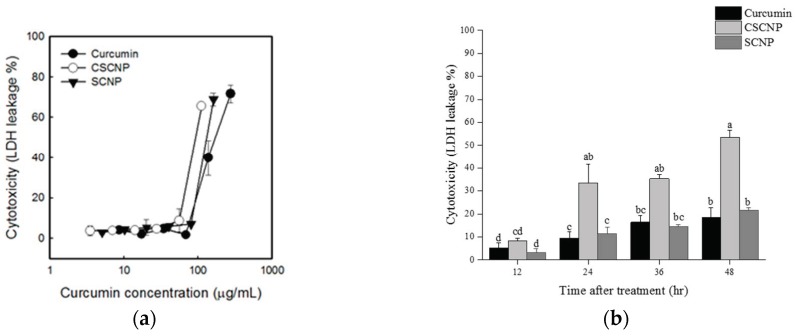
Lactate dehydrogenase (LDH) leakage of Hep G2 after different curcumin, or nanoparticle dosages and time durations. The cells were treated with samples for (**a**) 48 h at different dosage and (**b**) 100 μg/mL at different times. The initial cell number was adjusted to 2 × 10^5^ cells/mL. Data were shown by mean ± SD of three independent experiments (*n* = 3) with three technical replicates. LDH: lactate dehydrogenase; SCNP: silica-encapsulated curcumin nanoparticles; CSCNP: chitosan with silica co-encapsulated curcumin nanoparticles.

A higher concentration of lactate dehydrogenase LDH is present in tumor cells. The cytotoxicity of drugs was determined by evaluating the amount of LDH released from the damaged tumor cells [45]. DNA fragmentation is also associated with apoptosis during cancer therapy [46]. An LDH leakage assay was performed to analyze the cytotoxicity of curcumin and curcumin nanoparticles against Hep G2 cells. Figure 5a shows that all samples increased the percentage of LDH leakage at a concentration between approximately 70~80 μg/mL. The cytotoxicity of each sample increased with longer duration. A significant increase was observed in CSCNP treated cells at a lower curcumin concentration. In addition, more than 50% of LDH leakage was observed in CSCNP treated groups. This result supports the MTT assay and apparently CSCNP was more cytotoxic against Hep G2 cells. 

### 2.6. Storage Test

Curcumin is unstable due to its specific chemical and physical properties. The cell viability test (MTT assay) and anti-oxidation test were used to determine the stabilities of the particles. It was found that the effects of curcumin and nanoparticles on cell cytotoxicity were decreased with increasing storage time. Curcumin show a dramatic increase in the IC_50_ of the cell survival rate after 80 days of storage, while the CSCNP and SCNP only showed a slight increase. After the 80 days’ storage, the efficiency defined in Table 3 of the curcumin was 28.9%, but both CSCNP and SCNP showed more than 80% of efficiency.

**Figure 6:**
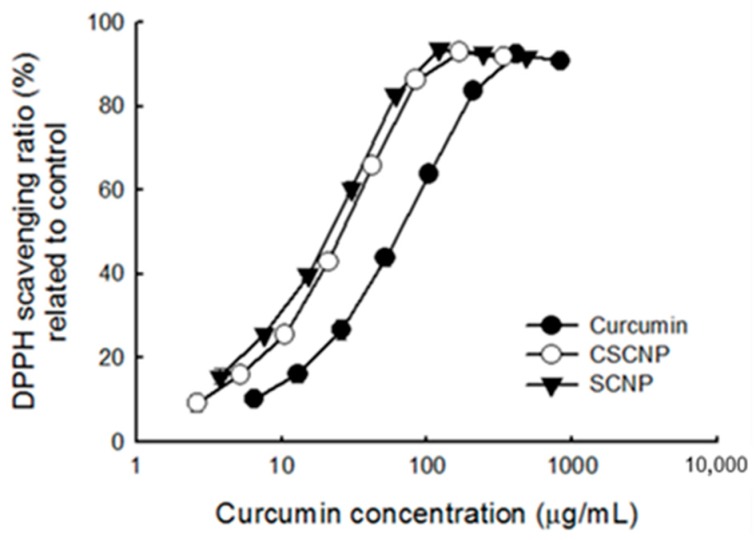
DPPH scavenging ability of curcumin, SCNP, and CSCNP after irradiated with an ultraviolet (UV-C) 30W lamp overnight (about 10 h). Data were shown by mean ± SD of 3 independent experiments (*n* = 3) with 3 technical replicates. DPPH: 2, 2-diphenyl-1-picrylhydrazyl; SCNP: silica-encapsulated curcumin nanoparticles; CSCNP: chitosan with silica co-encapsulated curcumin nanoparticles.

The oxidation resistance of samples after irradiated with an ultraviolet (UV) lamp was also determined. In the anti-oxidation test, the samples were simultaneously irradiated with an ultraviolet light tube (UV-C, 30 W) in an aseptic workstation (over 10 h), and followed by a DPPH radicals scavenging test. It was found that the UV-irradiated curcumin significantly reduced its antioxidant capacity (Figure 6) as compared to SCNP and CSCNP. The efficiency of both SCNP and CSCNP were higher than curcumin. The decrease in the ability of curcumin to scavenge DPPH free radicals is mainly due to the decomposition of curcumin by UV light. However, both SCNP and CSCNP showed more than 100% efficiency (Table 4)

## 3. Discussion

*Curcuma longa* (turmeric) belongs to the family Zingiberaceae and has been well-known for its effect on treating inflammatory and other diseases. Curcumin (diferuloylmethane) is a less toxic polyphenol derived from *Curcuma longa* with chemical formula of (1, 7-bis (4-hydroxy-3-methoxyphenyl)-1,6-heptadiene-3,5-dione) [47]. Curcumin acts as a potent scavenger for a variety of reactive oxygen species (ROS), inhibits lipid peroxidation, and reduces oxidative cell injury etc. [48]. Previous studies showed that curcumin down-regulates cyclooxygenase-2, inhibits nuclear factor kappa B (NF-κB) expression and reduce tumor necrosis factor (TNF)-α expression. The anti-cancer effect of curcumin is achieved by the inhibition of cell cycle progression and the induction of apoptosis. It blocks the inhibition of protein tyrosine kinase and c-myc messenger ribonucleic acid (mRNA) expression. Curcumin damages the DNA and impairs the ubiquitin-proteasome system through the mitochondrial pathway and thereby promote apoptosis. Curcumin causes a rapid decrease in mitochondrial membrane potential and release of cytochrome c to activate caspase 9 and caspase 3 for apoptotic cell death [49]. MTT assay in A549 cells showed that the curcumin (CM)-loaded nanoparticles exhibited better cytotoxicity with higher number of apoptotic bodies than free CM at * *p* < 0.05. This was due to the increased intracellular uptake of nanoparticles by cells [50].

The direct use of curcumin is limited due to low water solubility, low bioavailability, chemical instability, rapid metabolization within the gastrointestinal tract (GIT), intense color, and strong flavor [35]. In order to overcome this problem, nanotechnology-based encapsulation methods are employed. Inorganic particles incorporated biomolecules exhibit improved properties of the drug. Mesoporous silica nanoparticles (MSN) have characteristics of tunable porosity and size, biocompatibility, and high surface area. The modification of MSN can control cellular uptake, drug release, and endosomal escape [51]. Chitosan is a biopolymer obtained by the deacetylation of chitin. It was shown that chitosan is able to control drug release, enhance efficiency, improves drug solubility and stability, and reduces toxicity [52]. Silica-encapsulated curcumin nanoparticles (SCNP) and chitosan with silica co-encapsulated curcumin nanoparticles (CSCNP) were prepared by silicification and biosilicification methods, respectively. Chitosan-mediated formation of biomimetic silica nanoparticles involving the hydrolysis/condensation and aggregation of silica source by the terminal amine groups of chitosan [11]. From SEM analysis, the average size of CSCNP and SCNP were 75.0 ± 14.62 nm and 61.7 ± 23. 04 nm, respectively. It was also observed that the particles were in a spherical shape (Figure 1). The particle size was confirmed by DLS analysis and the average particle sizes of SCNP and CSCNP were bigger than that analyzed with SEM (Table 1). The size variation was due to the different analysis methods. For SEM, the particles were analyzed in a dry form whereas in DLS analysis the samples were dissolved in water. The particle size was reduced in SEM due to the particle shrinkage because of the loss of moisture content. A previous study reported that the biocompatibility of silica can be improved in the presence of chitosan [53]. The small size, leaky vasculature, and EPR effect enable nanoparticle to accumulate in the body and then internalized into the cells via endocytosis [54]. 

DPPH radical scavenging and ferrous ion chelating activity were performed to evaluate the antioxidant activities of SCNP and CSCNP. Antioxidants reduce cell damage by decreasing the formation of reactive oxygen species (ROS) [55]. Free radical-mediated lipid peroxidation, DNA damage, and production of ROS are the key mechanisms exerted by curcumin to reduce tumor cell growth [56]. Studies showed that the ER stress, intracellular Ca^2+^, and ROS production were increased after treatment with curcumin [57]. 2,2-diphenyl-1-picrylhydrazyl (DPPH) is a stable free radical with deep violet color. If the free radicals have been scavenged, the color will be changed to yellow. The hydrogen atom from the antioxidant reduces the odd electron of the nitrogen atom in DPPH [58]. It was understood that curcumin, SCNP, and CSCNP have DPPH radical scavenging activity and CSCNP showed slightly better activity than others (Figure 2b). The formation of free radicals by gain or loss of electrons is achieved by the transition metal ion Fe^2+^ and the reduction of ROS production by the chelation of metal ions with chelating agents [59]. Only SCNP and CSNP showed ferrous ion chelating activity at higher concentration. The better antioxidant activity of CSCNP may due to the participation of hydroxyl groups (-OH) and amino groups (NH_2_) from chitosan. The donation of hydrogen or the lone pairs of electrons enables the chitosan to chelate metal ions or scavenge free radicals [60]. The increased antioxidant activity of curcumin when loaded to nanoparticles were shown by DPPH scavenging activity in the confidence interval of 99% (*p* < 0.01) [61]. The combined antioxidant activity of curcumin and chitosan were shown by Fan et al., 2017. The DPPH scavenging activity was 249 13.2 % for curcumin loaded chitosan (CS) and 43.8 % was for curcumin loaded chitosan-chlorogenic acid (CS-CA) at 10 μg/mL. It was reported that mechanism for antioxidant activities of CS is mainly due to the hydrogen-donating ability [62].

An MTT assay was carried out to analyze the cytotoxicity of curcumin, SCNP, and CSCNP towards the tumor cell lines such as human melanoma cell line A375, human lung carcinoma cell line A549, human cervical squamous carcinoma cell line HeLa, human hepatoma cell line Hep G2, human colon carcinoma cell line HT-29, human breast carcinoma cell line MCF-7, and human gastric adenocarcinoma cell line MKN-28. The MTT assay is a very useful method to determine the toxicity of chemotherapeutics to the tumor cells. The number of living cells is proportional to the amount formazan (dark blue) produced from MTT (yellow) by mitochondrial dehydrogenase enzymes in living cells. IC_50_ is a concentration of the tested drug able to cause the death of 50% of the cells. More cytotoxicity of the substance is denoted by a lower IC_50_ value [63]. The literature showed that the effect of curcumin varied from cell to cell and the cellular uptake of curcumin in cancer cells was higher than the normal cells [41]. Another important aspect is based on the level of glutathione. Studies showed that the low level of glutathione in cancer cells makes them more sensitive towards curcumin [42]. It was also noted that the high expression of NF-κB in cancerous could reduce after being treated with curcumin [41]. The good biocompatibility and biodegradation of both chitosan and silica were described elsewhere [64,65]. MSN has tunable pore size with good chemical and thermal stability but exerts certain toxicity at high doses. So, the addition of biopolymer chitosan will reduce the toxic nature of silica. In addition, the presence of amine groups make them pH-sensitive [66]. The presence of the acidic environment of tumor cells facilitates the swelling of chitosan and enables the easy release of the drug [66,67]. The cytotoxicity of each sample is shown in Figure 3, and it was noted that the curcumin, SCNP, and CSCNP showed significant cytotoxicity towards all tumor cell lines and the toxicities increased with the increasing the concentrations of samples. Studies showed that the curcumin induced different mechanisms of action in each cancer cells. Literature revealed that the curcumin-induced G2/M arrest, inhibiting the assembly dynamics of microtubules, and suppressed the expression of *zeste homolog 2* (*EZH2*) gene in breast cancer cell line MCF-7 [68]. Wu et al., pointed out that curcumin induces apoptosis in human non-small cell lung cancer NCI-H460 cells through the endoplasmic reticulum (ER) stress and caspase cascade and mitochondrial-dependent pathways [57]. HeLa cells were more sensitive to the samples, whereas MCF-7 and MKN-28 were more tolerant to the samples. The IC_50_ values of CSCNP were lower than that of the curcumin and SCNP. The highest difference in the percentage of IC_50_ value was observed in Hep G2 cells (Table 2). Therefore, further analysis was conducted based on Hep G2 cells. Previous studies also show that both silica and curcumin nanoparticles have the ability to produce cytotoxicity towards Hep G2 cells [69,70]. A previous study reported the significant reduction of cancer cells by curcumin nanoparticle as compared to free curcumin. It suggested the internalization and localization of drug-loaded nanoparticles into the cancer cells [71]. Hepatocellular carcinoma is known the cancer that causes the third most deaths worldwide [72]. HepG2 cells are used as a model for liver cancer because of the wild apoptotic p53 gene, high expression of COX-2, phenotypically more hepatocytic than others, and expresses many differentiated essential hepatic functions [73]. The cytotoxicity of curcumin, SCNP, and CSCNP were particularly evaluated in Hep G2 cells using MTT assay in both dose-dependent and time-dependent manners. The viability of Hep G2 cells was gradually dropped after curcumin, SCNP, and CSCNP treatment. CSCNP showed higher cytotoxicity towards Hep G2 cells at a lower concentration (Figure 4a). After 48 h, the cytotoxicity caused by CSCNP was significantly higher than both curcumin and SCNP. Less than 20% of cell viability was observed in CSCNP treated cells (Figure 4b). The dose-dependent relationship of curcumin with the cell viability of Hep G2 cells were reported by Wang et al., in 2011 [44]. They suggested that curcumin altered the cell morphology and promoted apoptosis by triggering pro-apoptotic factors [44]. Recent literature showed that the selenium nanoparticle-coated curcumin induced intracellular ROS production, activated p53, and induced AKT signal pathway [72]. The nanoparticles enter into the cells via endocytosis and the size, shape, stiffness, and surface properties of the nanoparticles will influence the uptake [74]. The nanoparticles are retained in the blood for an appropriate time. Because they are not small enough to be excreted by the kidney and not large enough to be recognized by the reticuloendothelial system (RES). Due to the enhanced permeation and retention effect (EPR), nanoparticle will enter into the tumor cells through leaky vasculature and retained due to reduced lymphatic drainage [75]. As a result, the leaky vascular nature of cancerous cells allows the uptake of more nanoparticles rather than normal cells. Studies reported that curcumin arrests cell growth at G2/M phase and induces apoptosis in the human hepatoma cell line HepG2. The compartmental lipophilic properties of curcumin allow them to localized in the cell membrane. Fluorescent microscopic images of free curcumin and curcumin nanoemulsions on HepG2 cells have shown that the intensity of curcumin faded significantly with time, while the nanoemulsion showed a high intensity after 24 h. This confirmed the gradual release of curcumin from nanoemulsion [76]. LDH is a stable cytoplasmic enzyme that is released from the cells when the plasma membrane is damaged. The LDH release from the cells during apoptosis or necrosis is quantified by measuring the NADH production during the conversion of lactate to pyruvate. This NADH is responsible for the reduction of a tetrazolium salt into formazan [77]. In our study, the LDH leakage in Hep G2 cells was estimated both in dose-dependent and time-dependent manners. The LDH leakage was increased at concentrations between approximately 70~80 μg/mL, indicated that higher concentration of samples can act as an apoptotic inducer. In the case of CSCNP, the LDH leakage was increased with longer duration, which was significantly higher than both curcumin and SCNP. From the results, it was apparent that the curcumin and curcumin nanoparticles promote the apoptosis in cancer cells and it was confirmed by MTT and LDH leakage assays (Figure 5). These results agreed with a previous study in which the cell viability decreased with higher concentrations of the silica nanoparticles on cancerous cells through inducing cell membrane damage [53]. In cancer cells, the formation of lactate occurred due to the conversion of aerobic conditions to anaerobic conditions (Warburg effect). Literature indicated that LDH plays a crucial role in Warburg effect [78]. LDH convert pyruvate to lactate under anaerobic condition and identified as a biomarker of glycolytic activity. Tumor invasion, initiation, metastasis, and recurrence are associated with LDH and lactate production [79]. 

During apoptosis, the condensation of cytoplasm and plasma membrane blebbing lead to the breakdown of nuclear DNA. The chromosomal DNA was cleaved into multiples of ∼200bp oligonucleosomal size fragments during apoptosis [80]. The DNA fragmentation was assessed by electrophoresis. Figure S1 indicated that curcumin and curcumin nanoparticles promote apoptosis of Hep G2 cells by stimulating the DNA fragmentation and CSCNP caused more amount of DNA fragmentation than curcumin and SCNP. The expression of death receptor 5 (DR5) is an indicator of cell apoptosis. DR5 and/or DR4 promote cell death via TNF-related apoptosis-inducing ligand (TRAIL). Dominant-negative mutation in DR4 or DR5 that inhibits the apoptosis pathway is one of the characteristics of cancerous cells [81]. Because of the presence of a large number of a decoy receptors, TRAIL-mediated apoptosis does not cause toxicity to normal cells. Previous studies showed that curcumin promotes the upregulations of DR5 accompanying ROS generation and makes cells more sensitive to the cytotoxic activity of TRAIL [82]. Figure S2 showed that the DR5 expression in curcumin-treated cells were higher than that of nanoparticles. The lower expression of DR5 in Hep G2 cells may be due to the fact that nanoparticles do not interfere with the DR5 involved pathway. Further studies such as DR5 downstream event analysis or changes of sensitivity of the receptor and caspase assay (DNA fragmentation) are needed to confirm the activity of nanoencapsulated curcumin on cancer cells. 

Recent literature showed that the chemical instability, low bioavailability, and poor water dispersibility of curcumin have been improved by encapsulation [83]. The stability and efficiency of curcumin and nanoparticles were determined via MTT assay and an anti-oxidation test. After 80 days of storage, the IC_50_ values of curcumin increased significantly but nanoparticles showed only a slight difference on day 0. The efficiencies of both SCNP and CSCNP were higher than 80% indicated the stability of the particles was still acceptable after 80 days. After irradiation with UV light, the antioxidant activity of curcumin was reduced. The decrease in the ability to scavenge DPPH free radicals is mainly due to the decomposition of curcumin by UV light. The DPPH scavenging EC_50_ efficiencies of SCNP and CSCNP were significantly higher than that of curcumin. It is concluded that nanoencapsulation improves the instability of curcumin and helps to stimulate its anti-oxidant and antitumor properties via promoting cell membrane leakage and DNA damage.

Curcumin is generally recognized as a safe material by the Food and Drug Administration (FDA) and are nontoxic, non-mutagenic, and non-genotoxic in nature [84]. The pathways involved in the anti-tumor activity of curcumin may originate from cyclin-dependent, (b) p53-dependent and (c) p53-independent pathways [85]. Literature indicated that the effect of curcumin in cancer cells and normal cells are different. A low level of glutathione and a high level of NF-κB in cancer cells make them more sensitive towards curcumin [86]. The neovasculature of the tumor cell is characterized by impaired vessel and widespread blood vessel. Consequently, the leaky vascular nature of tumor cell provides the easy entry for the nanoparticles to move across cell membrane [87]. It was observed that both SCNP and CSCNP induced potential cytotoxicity to several cancer cell line particularly, to Hep G2 cells (demonstrated in Table 2). The cytotoxicity of nanoparticles was significantly higher than the pure curcumin. Our results demonstrated that nanoencapsulation of curcumin with silica and chitosan not only increase curcumin stability but also enhance cytotoxic activity and LDH leakage on hepatocellular carcinoma cells. This study provided initial data regarding potential cytotoxic activity of SCNP and CSCNP in different cancer cell lines and can be considered as a novel drug delivery system for increasing the bioavailability of curcumin, 

## 4. Materials and Methods

### 4.1. Materials

Human melanoma cell line A375, human lung carcinoma cell lineA549, human cervical squamous carcinoma cell line HeLa, human hepatoma cell line Hep G2, human colon carcinoma cell line HT-29, human breast carcinoma cell line MCF-7, and human gastric adenocarcinoma cell line MKN-28 were purchased from the American Type Culture Collection (ATCC) and Dr. Murakami’s Research Laboratory, Kyushu University, Japan. Dulbecco’s modified Eagle’s medium (DMEM) and trypsin- ethylenediaminetetraacetic acid (EDTA) were obtained from Invitrogen, California, USA. Fetal bovine serum (FBS) was purchased from HyClone (Logan, UT, USA). Aquaresin Turmeric was purchased from KALSCE®, Michigan, USA. Sodium silicate solution was obtained from Wako Pure Chemical, Osaka, Japan. Chitosan was obtained from LYTONE Enterprise. Inc., Taipei, Taiwan. 2,2-diphenyl-1-picrylhydrazyl (DPPH) and 3-(4,5-dimethylthiazol-2-yl)-2,5-diphenyltetrazolium bromide (MTT) were purchased from Sigma Aldrich, Missouri, USA. Lactate dehydrogenase (LDH) leakage kit was acquired from Promega, Wisconsin, USA. PE-conjugated anti-human DR5 was purchased from ebioscience, San Diego, CA, USA. Ferrous chloride and ferrozine (3-(2-pyridyl)-5,6-diphenyl-1,2,4-triazine-4’,4’’-disulfonic acid sodium salt) were purchased from Sigma, St. Louis, MO, USA.

### 4.2. Methods

#### 4.2.1. Cell Culture

All cells (except human gastric adenocarcinoma cell line MKN-28) were cultured in Dulbecco’s modified Eagle’s medium (DMEM) supplemented with 10% FBS at 37 °C in a 5% CO_2_ incubator. Roswell Park Memorial Institute (RPMI) 1640 Medium was used for human gastric adenocarcinoma cell line MKN-28. 

#### 4.2.2. Preparation of Nano-Encapsulated Curcumin

Chitosan samples were purchased from a commercial supplier were analyzed for the degree of deacetylation (DD) and molecular weight (Mw) according to previous reports [88]. The DD of chitosan samples were 90% with an Mw of 20kDa; 0.82% (*w/w*) sodium silicate solution was prepared by dissolving in 100 mL, 0.05 M sodium acetate buffer and stirred for 3 min [89]. Later, silica encapsulated curcumin nanoparticles (SCNP) were prepared by adding 10 mL curcumin solution to the above solution under strong agitation condition. The nanoencapsulated curcumin obtained was centrifuged (26,100× *g*) and freeze-dried. Chitosan with silica co-encapsulated curcumin nanoparticles (CSCNP) were obtained by stirring the mixture of sodium citrate solution, the chitosan solution, and curcumin solution (10:1:1) for three days. After centrifugation (26,100× *g*), the supernatants were discarded, dialyzed (one day), and freeze-dried [53].

#### 4.2.3. Characterization of Nano-Encapsulated Curcumin

The particle size was measured by using dynamic light scattering (DLS) method using Malvern 4700c submicron particle analyzer (Malvern Instruments, Malvern, Worcestershire, UK). 0.1 g of nanoencapsulated curcumin particles were dispersed in 50 mL deionized water and sonicated for 30 min before the analysis. Hitachi S-4800 scanning electron microscope (SEM) was used to observe the size and morphology of the nanoparticles. Lyophilized nanoparticles were transferred to carbon discs and coated with a gold layer at an accelerating voltage of 20 kV.

#### 4.2.4. 2,2-diphenyl-1-picrylhydrazyl (DPPH) Radical Scavenging Activity

100 μL of samples were added to 100 μL of freshly prepared 1 mM DPPH solution and stirred for 30 min at room temperature. The absorbance was measured at 517 nm. The concentration at which scavenged 50% (EC50) was determined by linear interpolation [90].

% DPPH inhibition = [1 − (A517_sample_/A517_blank_)] × 100 (1)

#### 4.2.5. Determination of the Ability to Chelate Ferrous Ions

0.5 mL of Methanol and 0.025 mL of 2 mM Iron (II) chloride were added to 0.5 mL of different concentrations of the samples. After 30 s, 0.05 mL of 5 mM ferrozine was added. After 10 min, the absorbance was measured at 562 nm using a spectrophotometer [91].

Chelating ratio % = [1 − (sample A562 − background value A562)/(control group A562 − background value A562)] × 100(2)

#### 4.2.6. Cell Viability Assay

2 × 10^5^ cells/mL (100 μL/well) were seeded into 96-well plate containing medium supplemented with 2% FBS and incubated in 5% CO_2_ incubator for overnight. Cells were treated with 20 μL of different concentrations of samples and incubated for 12–48 h. Later, the medium was aspirated from the wells and 120 μL of fresh medium was added. After 1 hour, 100 μL of the MTT solution (0.5 mg/mL) was added to the cells and incubated for 4 h. The absorbance was measured at 570 nm. The concentration at which cell growth was inhibited by 50% (the 50% inhibitory concentration (IC_50_) was determined by linear interpolation [(50% − low percentage)/(high percentage − low percentage)] × (high concentration − low concentration) + low concentration] [92].

Relative viability (%) = [A sample]/[A control] × 100(3)

where [A]sample and [A]control denote the absorbance of the sample and control, respectively [53]. 

#### 4.2.7. Lactate Dehydrogenase Leakage Assay

The CytoTox96 nonradioactive assay kit (LDH assay) was used to perform the LDH assay. Hep G2 cells were seeded in 96-well plates, exposed to samples, and incubated for 48 h. After incubation, the 96-well plates were centrifuged at 430× *g* for 5 min and the cell medium was transferred to another new 96-well plates (50 μL/well). After the addition of LDH substrate (50 μL/well), the plates were kept under a dark atmosphere for 30 min. 1N hydrochloric acid (HCl) (25 μL/well) was then added to each sample to terminate the reaction. The absorbance was measured at 490 nm. Control experiments were performed with 0.1% (*w/v*) Triton X-100 set as 100% cytotoxicity. 

LDH release was calculated by the following equation:

LDH (%) = ([A]sample − [A]medium)/([A]100% − [A]medium) × 100(4)

where [A]sample, [A]medium, [A] 100% denote the absorbance of the sample, medium control, and Triton X-100 control, respectively. All experiments were run in triplicate [53].

#### 4.2.8. DNA Fragmentation

Hep G2 cells were seeded into 10 cm dishes at a density of 2 × 10^5^ cells/mL (10 mL/dish) in medium supplemented with 2% FBS and incubated overnight. Different concentrations of nanoparticles were added to dishes (2 mL/dish) under the dark condition and incubated for 12–48 hours. After treatment, the cells were centrifuged at 250× *g*. Cells were lysed in a buffer containing10 mM Tris (pH 7.4), 150 mM NaCl, 5 mM EDTA and 0.5% Triton X-100 for10 min on ice. Lysates were vortexed and centrifuged at 14,000× *g* for 10 min. Fragmented DNA in the supernatant was extracted with an equal volume of neutral phenol: chloroform: isoamyl alcohol mixture (25:24:1) and analyzed electrophoretically on 2% agarose gels. Later, stained with ethidium bromide, and imaged with a FluoroImager (Pharmacia Biotech, D & R, Israel) [82].

#### 4.2.9. Analysis of Cell Surface Death Receptor 5 (DR5)

The cell number was adjusted to 2 × 10^5^. After the incubation with samples for one day, the cells were detached with 0.5 mM EDTA and washed three times with phosphate-buffered saline (PBS) wash buffer supplemented with 0.5% bovine serum albumin (BSA). Cells were resuspended in 200 μL of PBS, stained with the PE-conjugated anti-human DR5 (ebioscience, San Diego, CA) antibody (1 μg/mL) and incubated for 30 min at 4 °C. The unreacted antibody was removed by washing the cells twice with the same PBS buffer. Cell surface expression of the DR5 receptor was determined by flow cytometry (FACscan, BD Biosciences, Franklin Lake, NJ, USA). Fluorescent intensity of the cells is directly proportional to the density of receptor [82].

#### 4.2.10. Storage Test

Curcumin was dissolved in dimethyl sulfoxide (DMSO, Sigma-Aldrich Company, St. Louis, MO, USA) and nano-encapsulated curcumin was dissolved in distilled-deionized water. The final volume was adjusted to 10 mL and the concentration of DMSO was 0.1%. The samples were stored in a moisture proof cabinet and protected from light for 80 days. The cell viability and DPPH radical scavenging activity were analyzed according to Section 4.2.4 and Section 4.2.6, respectively.

### 4.3. Statistical Analysis

Data were expressed as means ± SD and analyzed using Student’s *t*-test of Sigma Plot 9.

## Figures and Tables

**Table 1:** Particle sizes analyzed by SEM and DLS.

	CSCNP	SCNP
SEM	75.0 ± 14.62	61.8 ± 23.04
DLS	112.88 ± 3.00	111.05 ± 2.95

Data were shown by mean ± SD of 3 independent experiments (*n* = 3) with 3 technical replicates. SEM: scanning electron microscopy; DLS: dynamic light scattering; SCNP: silica-encapsulated curcumin nanoparticles; CSCNP: chitosan and silica co-encapsulated curcumin nanoparticles.

**Table 2:** Comparisons of IC_50_ (μg/mL) of curcumin, SCNP, and CSCNP toward seven cancer cell lines.

Cell line	Curcumin	CSCNP	Difference ^a^	SCNP	Difference ^b^
**A375**	93 ± 3	53 ± 1	43%	65 ± 1	30%
**A549**	98 ± 0	56 ± 4	43%	81 ± 2	17%
**HeLa**	68 ± 4	42 ± 0	38%	56 ± 1	18%
**Hep G2**	90 ± 2	46 ± 3	49%	41 ± 10	54%
**HT-29**	106 ± 2	62 ± 0	42%	72 ± 3	32%
**MCF-7**	153 ± 7	80 ± 0	48%	112 ± 1	27%
**MKN-28**	166 ± 9	89 ± 1	46%	135 ± 1	19%

Differences were shown as the percentage decrease in IC_50_ after nanoencapsulation. SCNP: silica-encapsulated curcumin nanoparticles; CSCNP: chitosan with silica co-encapsulated curcumin nanoparticles. Difference ^a^ and difference ^b^ were calculated by the following formulas: Difference ^a^ = 100 − (CSCNP IC_50_/Curcumin IC_50_) × 100; Difference ^b^ = 100 − (SCNP IC50/Curcumin IC_50_) × 100.

**Table 3:** Comparisons of the cell viability IC_50_ of curcumin, SCNP, and CSCNP in Hep G2 cells while stored in water for 80 days (μg/mL).

Sample Name	Storage at 0 Day	Storage after 80 Days	Efficiency ^a^ (%)
Curcumin	72	251	28.9
CSCNP	111	127	87.4
SCNP	140	166	84.3

^a^ Formula = 1/(Storage after 80 days IC_50_/Storage at 0 day IC_50_) × 100. SCNP: silica-encapsulated curcumin nanoparticles; CSCNP: chitosan with silica co-encapsulated curcumin nanoparticles.

**Table 4:** Comparisons of DPPH scavenging EC_50_ of curcumin, SCNP, and CSCNP while with and without UV irradiation (μg/mL).

Sample Name	Irradiated at 0 Hour	Irradiated at Overnight	Efficiency ^a^
Curcumin	59	68	86.8%
CSCNP	32	28	114.3%
SCNP	44	23	191.3%

^a^ Formula = 1/(Irradiated overnight EC_50_/Irradiated 0-h EC_50_) × 100. EC_50_: half maximal effective concentration SCNP: silica-encapsulated curcumin nanoparticles; CSCNP: chitosan and silica co-encapsulated curcumin nanoparticles.

## Supplementary Materials

Supplementary materials can be found at https://www.mdpi.com/1422-0067/20/12/2918/s1.

## Abbreviations

SCNP	silica encapsulated curcumin nanoparticles
CSCNP	chitosan with silica co-encapsulated curcumin nanoparticles
MSNs	mesoporous silica nanoparticles
EPR	enhanced permeability and retention effect
ROS	reactive oxygen species
DR5	death receptor 5
DISC	death-inducing signaling complex
