# Supplementary material for: Curcumin-Loaded Mesoporous Silica Nanoparticles Markedly Enhanced Cytotoxicity in Hepatocellular Carcinoma Cells
Source: Int J Mol Sci. 2019 Jun 14;20(12):2918. doi: 10.3390/ijms20122918 (PMC6628080; doi:10.3390/ijms20122918)
Supplement: Supplementary file 1 [file ijms-20-02918-s001.pdf]

Supplementary data

Deoxyribonucleic acid (DNA) Fragmentation

DNA fragmentation is one of the features of apoptosis. Upregulation of endonuclease, intranuclear/intracellular redistribution, and primary changes of chromatin structures are the major pathways of DNA fragmentation during apoptosis. After 24 h of treatments, the DNA was extracted for determining the fragmentation of DNA using electrophoresis. From figure S1, it was indicated that curcumin and nanoparticles cause DNA fragmentation and the fragmentation was increased with the increase in concentrations of the samples. As compared to curcumin and SCNP, the CSCNP better stimulated the apoptosis of the Hep G2 cells via increasing DNA fragmentation.

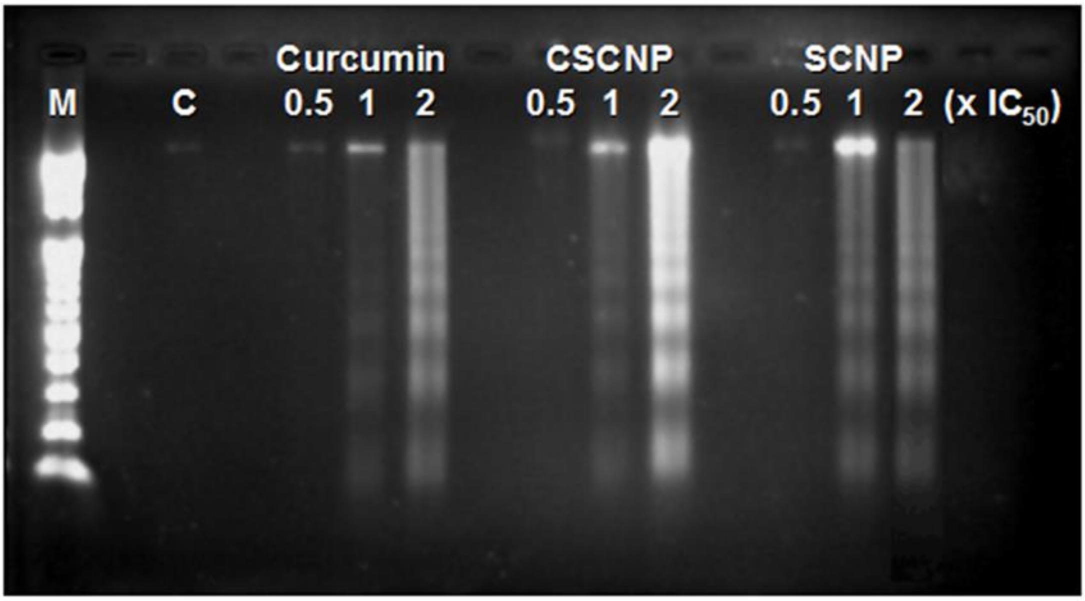

Figure S1. Hep G2 DNA fragmentation of curcumin, SCNP, and CSCNP. The cell number were adjusted to 2 × 10^5 cells/mL and treated with samples for 24 h. The concentrations of curcumin, CSCNP and SCNP were 0.5, 1 and 2 folds of IC50. M: DNA marker, C: control; SCNP: silica-encapsulated curcumin nanoparticles; CSCNP: chitosan with silica co-encapsulated curcumin nanoparticles.

Cell surface DR5 expression

DR 5 have a selective ability to kill tumor cells. It promotes the apoptosis via recruiting caspase-8 and caspase-10, thereby forming the death-inducing signaling complex (DISC) [1]. The effects of curcumin (Figure S2a), CSCNP (Figure S2b) and SCNP (Figure S2c) on the expression of DR5 on cancer cells were analyzed by the flow cytometry. It was found, CSCNP and SCNP did not increase the expression of DR5 on cancer cells and that was significantly lower than the curcumin.

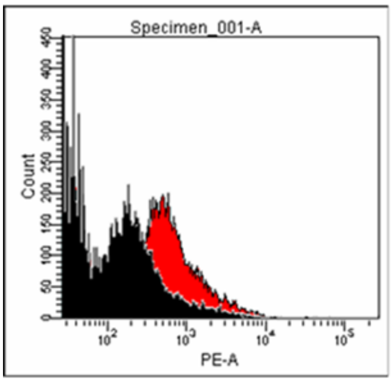

(a)

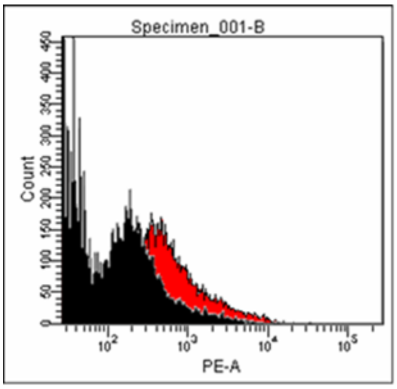

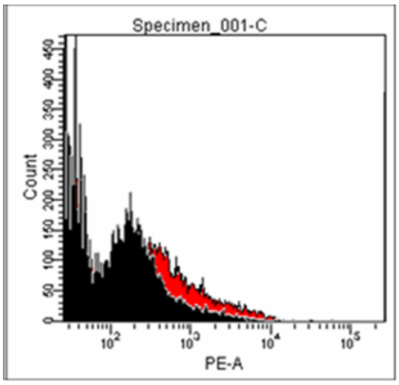

(b) (c)

Figure S2. Hep G2 cell surface DR5 expression assay by the flow cytometer after treated with (**a**) Curcumin, (**b**) CSCNP, and (**c**) SCNP. Hep G2 cells (5 × 105) were treated with samples for 24 hours and stained with PE-conjugated DR5 antibody and analyzed using the flow cytometer. Data acquired were based on 105 cells per sample. SCNP: silica-encapsulated curcumin nanoparticles; CSCNP: chitosan with silica co-encapsulated curcumin nanoparticles.

References

1. Li, J.; Niu, R.; Dong, L.; Gao, L.; Zhang, J.; Zheng, Y.; Shi, M.; Liu, Z.; Li, K. Nanoencapsulation of curcumin and its protective effects against CCl_4-induced hepatotoxicity in mice. *J. Nanomater.* 2019, *2019*, doi:10.1155/2019/7140132.
